# The Combination of Long-term Ketamine and Extinction Training Contributes to Fear Erasure by Bdnf Methylation

**DOI:** 10.3389/fncel.2017.00100

**Published:** 2017-04-20

**Authors:** Ling-Sha Ju, Jiao-Jiao Yang, Lei Lei, Jiang-Yan Xia, Dan Luo, Mu-Huo Ji, Anatoly E. Martynyuk, Jian-Jun Yang

**Affiliations:** ^1^Department of Anesthesiology, Zhongda Hospital, Medical School, Southeast UniversityNanjing, China; ^2^Department of Anesthesiology and the McKnight Brain Institute, University of Florida College of MedicineGainesville, FL, USA

**Keywords:** ketamine, extinction, inescapable foot shocks, DNA methylation, BDNF

## Abstract

A combination of antidepressant drugs and psychotherapy exhibits more promising efficacy in treating fear disorders than either treatment alone, but underlying mechanisms of such treatments remain largely unknown. Here we investigated the role of DNA methylation of the brain-derived neurotrophic factor (Bdnf) gene in the therapeutic effects of ketamine in combination with extinction training in a mouse model of post-traumatic stress disorder (PTSD) induced by inescapable electric foot shocks (IFS). Male mice received ketamine for 22 consecutive days starting 1 h after the IFS (long-term ketamine treatment) or 2 h prior to the extinction training on days 15 and 16 after the IFS (short-term ketamine treatment). The Open Field (OF) and Elevated Plus Maze (EPM) tests were conducted on days 18 and 20. The spontaneous recovery and fear renewal tests were performed on day 23. Mice, subjected to IFS, exhibited anxiety-like behavior and fear relapse, accompanied by the increased levels of DNA methyltransferases, hyper-methylation of Bdnf gene, and decreased BDNF mRNA expression in the medial prefrontal cortex (mPFC) and hippocampus (HIP). Long-term treatment with ketamine combined with extinction training alleviated the IFS-induced abnormalities. These results suggest that long-term ketamine treatment in combination with extinction training may ameliorate fear relapse in the murine model of PTSD, at least in part, by normalizing DNA methylation of Bdnf gene.

## Introduction

Posttraumatic stress disorder (PTSD), induced by a single or repeated exposures to severe traumatic events and/or physiological stress, frequently lead to long-lasting debilitating illnesses (Gustavsson et al., 2011; Bisson et al., 2015). These disorders not only seriously undermine the mental health of the affected individuals, but also impose an enormous public health and economic burden on society (Yehuda and LeDoux, 2007; Gustavsson et al., 2011).

Early attempts at combining cognitive behavior therapy with anxiolytic medications (e.g., benzodiazepines) showed that the combination was no more effective, in some instances even counter-productive, than psycho- or pharmacotherapy alone (Marks et al., 1993; Wilhelm and Roth, 1997). However, at least in some cases, this failure may have reflected idiosyncratic effects of the drugs tested (especially benzodiazepines) rather than utility of the strategy itself, and there has been an intense search to identify agents that serve as more effective adjuncts to cognitive behavior therapy (Hetrick et al., 2010; Hendriksen et al., 2014; Singewald et al., 2015). Moreover, there is evidence that the efficacy of different classes of drugs combined with the exposure therapy may be distinctly influenced by a number of factors including age, gender and other examples of individual differences (Bandelow et al., 2015). Therefore, there is an urgent need in elucidation of the neurobiological mechanisms underlying alleviating effects of combination of pharmaco- and psychotherapies.

Ketamine, a noncompetitive N-methyl-D-aspartate (NMDA) receptor antagonist, has been shown to exert antidepressant effects as rapidly as 2 h following a single sub-anesthesia doses injection to patients with major depressive disorder (Berman et al., 2000; Krystal et al., 2013; Monteggia and Zarate, 2015; Yang et al., 2015). Recently, ketamine has emerged as a potential therapeutic agent for PTSD. In a randomized clinical trial, intravenous infusion of ketamine hydrochloride (0.5 mg/kg) induced a rapid reduction of core PTSD symptoms when assessed 24 h after infusion (Feder et al., 2014). Also, lower incidence of PTSD was reported in soldiers treated with ketamine during their operations at a military medical center (McGhee et al., 2008). Based on these findings, ketamine could be a promising candidate to be used as pharmacological component for combined pharmaco- and psychotherapy for PTSD.

Brain-derived neurotrophic factor (BDNF) has been previously identified as a component of fear extinction mechanisms given that blunted activity of BDNF signaling was associated with deficient extinction retrieval in rats (Kabir et al., 2013). Conversely, increased levels of BDNF mRNA were observed in the prefrontal cortex (PFC) following successful fear extinction (Bredy et al., 2007). Also, the extinction-resistant female mice were found to exhibit increased DNA methylation of Bdnf (Baker-Andresen et al., 2013). This finding suggests that DNA methylation mediated gene expression could contribute to the mechanism underlying fear erasure. Importantly, sub-chronic ketamine administration has been shown to increase the expression of Bdnf gene in the rat brain (Duman and Monteggia, 2006; Autry et al., 2011; Liu et al., 2015).

By using an animal model of PTSD that consisted of 10 inescapable electric foot shocks (IFS), we investigated whether ketamine combined with extinction training may diminish anxiety-like behavior and facilitate persistent fear erasure in adult mice. The antidepressant fluoxetine (Flx) was studied as the positive control. In addition, we assessed whether DNA methylation of Bdnf gene could be involved in mediation of fear erasure caused by ketamine in combination with extinction training.

## Materials and Methods

### Animals

One-hundred and sixty-five male C57BL/6 mice (7–8 weeks) were purchased from the Animal Center of Jinling Hospital, Nanjing, China. This initial study was carried out in male mice in order to control for hormonal variables that are known to influence conditioned fear responding and extinction. The mice were housed five per cage in standard conditions with a 12-h light/dark cycle (light from 07:00–19:00) at 23 ± 1°C and* ad libitum* access to food and water. The present study was approved by the Ethics Committee of Jinling Hospital, Nanjing University, China, and the study was conducted in accordance with the Guideline for the Care and Use of Laboratory Animals from the National Institutes of Health, USA.

### PTSD Model and Extinction

#### Apparatus

The IFS apparatus consists of a brightly lit Plexiglas chamber (30 cm long × 26 cm wide × 22 cm high) placed in a ventilated, sound-attenuated box (XR-XC404; Shanghai Softmaze Information Technology Co., Ltd., Shanghai, China). The foot shock was delivered through stainless steel bars by a constant current generator (Med Associates, Inc., St. Albans, VT, USA). The tone was generated by a speaker and sound intensities were measured by an audiometer. The behavior of the mice was recorded using a digital video camera on the ceiling of the box.

#### Inescapable Electric Foot Shocks (PTSD Model)

Freezing behavior, defined as the absence of all visible movement of the body except the movement necessitated by respiration, is expressed as a percentage of time spent on freezing. IFS and extinction took place in two different contexts unless otherwise stated. IFS context A was transparent Plexiglas chamber with metal grids on floor whereas extinction context B was black nontransparent Plexiglas chamber with planar floor. Both context A and context B were cleaned with 75% ethanol before each session. After 7 days of accommodation, an animal model of PTSD was established according to the study described previously (Ressler et al., 2011). Briefly, mice were habituated to context A on day 1 and presented with 10 tone-shock pairings at an inter-trial interval (ITI) of 3–5 min after 5 min. Each pairing consisted of a 30 s tone (CS, 6 kHz, 85 db) that co-terminated with a 1 s, 1.0 mA foot shock (US). In the Vehicle (Veh) group mice received the same treatment without foot shocks.

#### Fear Extinction Training

On days 15 and 16 after the IFS mice were submitted to extinction training in context B during which they received 12 presentations of the CS only on each day (ITI: 20–60 s).

#### Spontaneous Recovery and Context Dependent Fear Renewal

Seven days after the second extinction training, the mice were tested in context B for spontaneous recovery and context A for fear renewal, respectively, by using four presentations of the CS (ITI: 20–60 s).

### Drug Treatment and Design

#### Fluoxetine

Mice received fluoxetine (Flx, BioTrend Chemicals, Destin, FL, USA) via drinking water in light-protected tubes. Flx was dissolved in tap water at a concentration of 0.08 mg/ml to achieve an approximately 10 mg/kg per day dosing and the solutions were prepared fresh every day. This protocol of chronic Flx administration can result in Flx plasma levels within the therapeutic range in humans (Rantamäki et al., 2007). The treatment continued through all behavioral sessions until sacrifice.

#### Ketamine

Mice in the long-term ketamine treatment group were administrated with ketamine (0.625, 1.25, or 2.5 mg/ kg; Gutian Pharmaceutical Company, Fujian, China) intraperitoneally (i.p.) beginning 1 h after the IFS and once daily for subsequent 22 consecutive days. Mice in the short-term ketamine treatment group were pretreated with ketamine (5, 10, or 20 mg/kg, i.p.) 2 h prior to the extinction training on days 15 and 16. The doses of ketamine were selected based on previous studies, in which repeated low-dose injections of ketamine have a clear anti-PTSD-like effect (Zhang et al., 2015), and pretreatment with ketamine induced persistent stress resilience (Brachman et al., 2015). Mice in the Veh groups received equal number of injections of saline (i.p.).

#### Design

**Figure 1:**
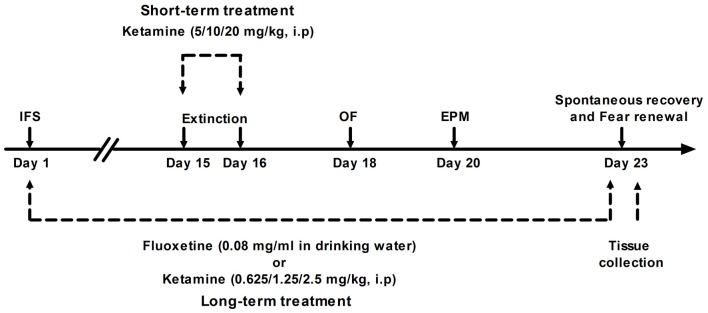
**Illustration of the experimental protocol**. See text for details. IFS, inescapable electric foot shocks; OF, open field test; EPM, elevated plus maze test.

The experimental design is shown in Figure 1. Mice were divided into long-term ketamine treatment group (*n* = 90) and short-term ketamine treatment group (*n* = 75). The first group of mice were equally randomized into six subgroups (*n* = 15 per subgroup): Veh group, IFS + Veh group, IFS + Flx group, IFS + Ket 0.625 (Ketamine 0.625 mg/kg) group, IFS + Ket 1.25 (Ketamine 1.25 mg/kg) group and IFS + Ket 2.5 (Ketamine 2.5 mg/kg) group. The second group was divided into five subgroups (*n* = 15 per subgroup): Veh group, IFS + Veh group, IFS + Ket 5 (Ketamine 5 mg/kg) group, IFS + Ket 10 (Ketamine 10 mg/kg) group and IFS + Ket 20 (Ketamine 20 mg/kg) group.

### Open Field (OF) Test

Explorative activities and anxiety-like behaviors were studied by using the OF apparatus (XR-XZ301; Shanghai Softmaze Information Technology Co., Ltd., Shanghai, China). The mice were gently placed in the center of a white plastic chamber (40 × 40 × 40 cm) and were allowed to explore the apparatus for 5 min and this autonomous exploration was automatically recorded by a video tracking system. The total distance traveled and the time spent in the center of the OF were recorded. At the end of each mouse test, the surface of the arena was cleaned thoroughly with 75% alcohol to avoid the presence of olfactory cues.

### Elevated Plus Maze (EPM) Test

The maze is a plus-cross-shaped apparatus consisting of four arms, two open and two enclosed by walls elevated 50 cm above the floor (XR-XG201; Shanghai Softmaze Information Technology Co., Ltd., Shanghai, China). Mice were placed in the center square facing an open arm and were allowed to explore the maze for 5 min. The percentage of time spent in and entries into the open arms was used as an anxiety index. Each mouse’s behavior was recorded using a video tracker.

### Tissue Harvest

There is considerable evidence indicating that the hippocampus (HIP) is critical for establishing contextual representations during fear conditioning and extinction (Fanselow, 2000; Heldt et al., 2007). However, it does not act alone, a growing literature has documented that the medial prefrontal cortex (mPFC) is critical for gating fear expression, in particular after extinction (Quirk and Mueller, 2008; Rozeske et al., 2015). Two hours after fear renewal, the mice were sacrificed, and the entire mPFC and HIP were dissected and stored at −80°C.

### Western Blot

Five mice in each subgroup were used for western blot. The mPFC and HIP were homogenized on ice in radioimmunoprecipitation assay buffer plus protease inhibitors (Sigma, St. Louis, MO, USA) and centrifuged at 4°C for 15 min at 12,000× rpm. The supernatant was then collected. The protein concentrations were measured according to the method of Bradford using bovine serum albumin as a standard (BioRad, Hercules, CA, USA). Protein extracts (60 μg/well) were separated by sodium dodecyl sulfate polyacrylamide gel electrophoresis (SDS-PAGE) on 8%–12% resolving gels and were then transferred to a polyvinylidene fluoride membrane (Millipore, Billerica, MA, USA). The membranes were blocked with 5% nonfat milk at room temperature for 1 h and then incubated at 4°C overnight with anti-DNMT1 (1:1000; Cell Signaling, Boston, MA, USA), DNMT3a (1:1000; Cell Signaling, Boston, MA, USA), anti- DNMT3b (1:1000; Abcam, Cambridge, UK), and anti-β-Actin (1:500; Abcam, Cambridge, UK). After washing, the membranes were incubated with horseradish peroxidase-conjugated goat anti-rabbit (1:1000; Santa Cruz Biotechnology, Dallas, TX, USA) or goat anti-mouse (1:1000; Bioworld Technology, Nanjing, Jiangsu, China). The bands were detected with Pierce ECL Western Blotting Substrate (Thermo Fisher Scientific, Rockford, IL, USA) and semi-quantified with ImageJ software (National Institutes of Health, Bethesda, MD, USA).

### Measurement of mRNA Levels by Real-time Reverse Transcriptase PCR (RT-PCR)

The mPFC and HIP from five mice in each subgroup were collected for RNA quantification. Total RNA was extracted using TRIzol (Life Technologies, Inc., Grand Island, NY, USA) following the manufacturer’s instructions. The mRNA was reverse transcribed using the First Strand cDNA Synthesis Kit (Thermo Fisher Scientific, Loughborough, UK). All primers were designed using Primer3 with BLAST sequence verification (see Table 1). RT-PCR amplifications were performed at 50°C for 30 min, 95°C for 15 min, followed by 40 cycles of 94°C for 60 s, 57°C for 60 s, 72°C for 60 s, and then incubation at 70°C for 10 min. PCR was performed in triplicate and threshold cycle numbers (CT) were averaged. Results were normalized to β-actin using the ∆∆CT method (Schmittgen and Livak, 2008).

**Table 1:** **Primers used in gene expression and DNA methylation assays**.

Type	Target gene	Primer sequence (5′-3′)
RT-PCR	Bdnf exon I	TTTTGGAGCGGAGCGTTTG
		TTTGCGGCTTACACCACCC
	Bdnf exon IV	CGCCATGCAATTTCCACTATCAATAATTTAAC
		CTTTTTCAGTCACTACTTGTCAAAGTAAAC
	Bdnf IX	GACCATCCTTTTCCTTACTATGG
		CCATTCACGCTCTCCAGAGTC
	β-actin	GTGACGTTGACATCCGTAAAGA
		GTAACAGTCCGCCTAGAAGCAC
Bisulfite	Bdnf exon IV	GAGGTAGAGGAGGTATTATATGATAG
DNA sequencing		CAAAATAAACATCAAAACAACTACT

### DNA Methylation Analysis

Bisulfite genomic conversion and sequencing analysis of individual clones were performed according to published protocols (Feng et al., 2010). Briefly, DNA was extracted from mPFC and HIP using TIANamp Genomic DNA Kit (TIANGEN Biotech Co., Ltd., Beijing, China) and treated with sodium bisulfite using EZ DNA Methylation-Gold^TM^ Kit (Zymo Research, CA, USA). The converted DNA were amplified by primers that amplify the same region of exon IV (The PCR primers are listed in Table 1), but independent of methylation status. The thermocycler protocol involved an initial denaturation cycle (5 min, 95°C), 50 cycles of denaturation (1 min, 95°C), annealing (1 min, 60°C) and extension (1 min, 72°C), followed by a final extension cycle (5 min, 72°C) terminating at 4°C, resulting in a 384 bp PCR product for exon IV. The PCR products were purified using a gel extraction kit (TIANGEN Biotech Co., Ltd., Beijing, China). pCR4.1 TOPO vector cloning was performed by following the manufacturer’s instructions (Invitrogen, Carlsbad, CA, USA). Normally 10 minipreps were set up per sample and individual clones were then sequenced to detect methylation sequencing patterns, and the methylation percentage was calculated by dividing methylated clones by total clones in the particular CpG sites.

### Statistical Analysis

Data are presented as mean ± standard error of the mean (SEM), and all analyses were performed using SPSS 17.0 software (version 17.0, IL, USA). Comparisons were analyzed by one-way analysis of variance (ANOVA) followed by Tukey *post hoc* test. Group comparisons in the weight were tested by two-way repeated measures ANOVA followed by Bonferroni test. *P* value less than 0.05 was considered statistically significant.

## Results

### Long-term, Rather than Short-term Ketamine Treatment, Combined with Extinction Training Attenuated Anxiety-like Behavior Induced by Inescapable Electric Foot Shocks

**Figure 2:**
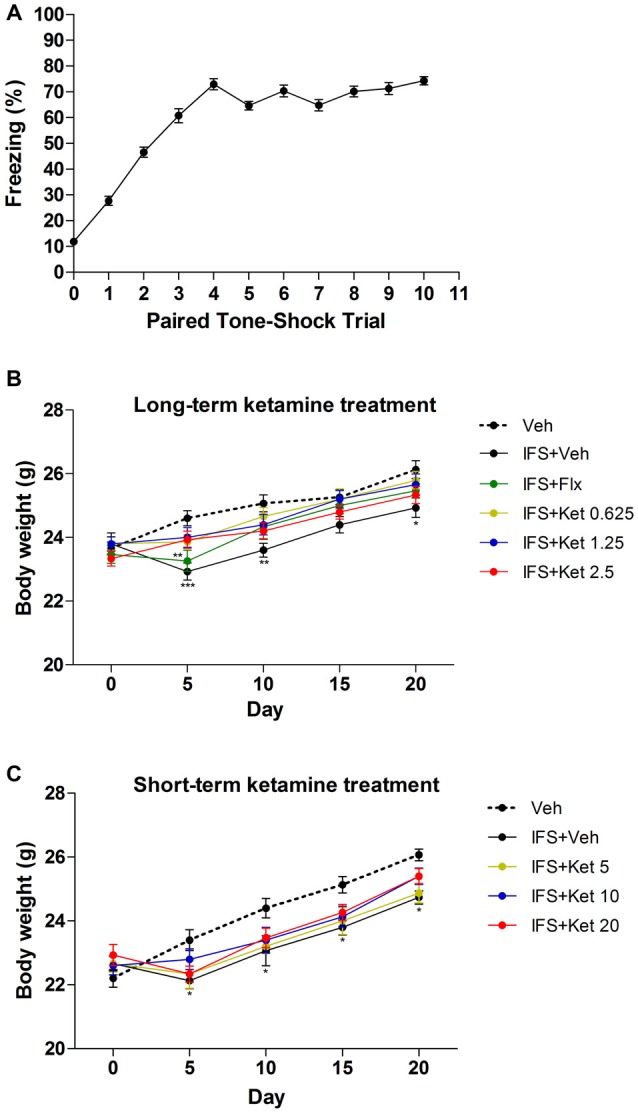
**The effects of ketamine treatment on body weight after electric foot shocks procedures. (A)** Percentage of time freezing, in mice, to the conditioned tone (CS) following tone-shock pairings during the conditioned fear trials. **(B,C)** IFS mice had lower body weight compared with the Veh group, and both Flx and ketamine alleviated the loss of body weight induced by IFS. There was no different in body weight among the varying doses of ketamine groups. Data are presented as mean ± SEM (*n* = 50 in **A**; *n* = 15 in per group). *P < 0.05, **P < 0.01, ***P < 0.001 compared with the Veh group.

Mice subjected to IFS exhibited progressively increased freezing time in response to each following CS reaching maximum at the 4th CS (1th CS: 27.706 ± 1.734; 4th CS: 72.569 ± 2.151; 5th CS: 64.620 ± 1.636; 6th CS: 70.333 ± 2.282; 7th CS: 64.783 ± 2.183; 8th CS: 7.138 ± 2.072; 9th CS: 71.329 ± 2.331; 10th CS: 74.302 ± 1.560, Figure 2A) and had reduced body weight (Day 5: *P* < 0.001; Day 10: *P* < 0.01; Day 20: *P* < 0.05 compared with the Veh group, Figure 2B; Day 5, 10, 15 and 20: *P* < 0.05 compared with the Veh group, Figure 2C). The effect of IFS on body weight was alleviated by Flx except on Day 5 (*P* < 0.01 compared with the Veh group) or ketamine.

**Figure 3:**
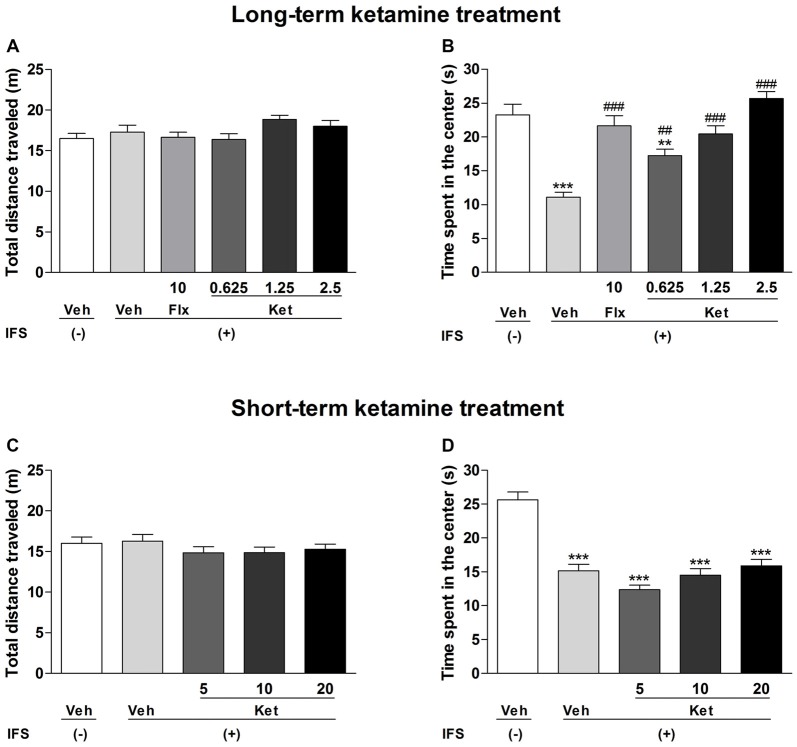
**The effects of ketamine on OF test in IFS mice. (A)** No significant difference was observed among the groups in the total distance traveled. **(B)** The IFS mice spent a significant shorter time in the center, which was significantly ameliorated in the groups that were administered either Flx or long-term ketamine treatment. **(C,D)** IFS mice manifested the normal locomotor activity, accompanying the shorter time spent in the center in the OF test, while short-term ketamine treatment didn’t alleviate the abnormalities. Data are presented as mean ± SEM (*n* = 15 in per group). **P < 0.01, ***P < 0.001 compared with the Veh group; ^##^*P* < 0.01, ^###^*P* < 0.001 compared with the IFS + Veh group.

In the OF test, there was no significant difference in the total distance traveled among all treatment groups (Figures 3A,C). However, IFS caused a significant decrease in the time spent in the center as compared with the Veh groups both in the long-term treatment (*F*_(5,84)_ = 18.142, *P* < 0.001, Figure 3B) and short-term treatment groups (*F*_(4,70)_ = 29.891, *P* < 0.001, Figure 3D). Long-term treatment with Flx or ketamine increased the time spent in the center in comparison with the IFS + Veh group (Flx: *P* < 0.001; ketamine (0.625 mg/kg): *P* = 0.008; ketamine (1.25 mg/kg and 2.5 mg/kg): *P* < 0.001, Figure 3B). The short-term administration of ketamine had no effect on the time spent in the center compared with the IFS + Veh group (Figure 3D).

**Figure 4:**
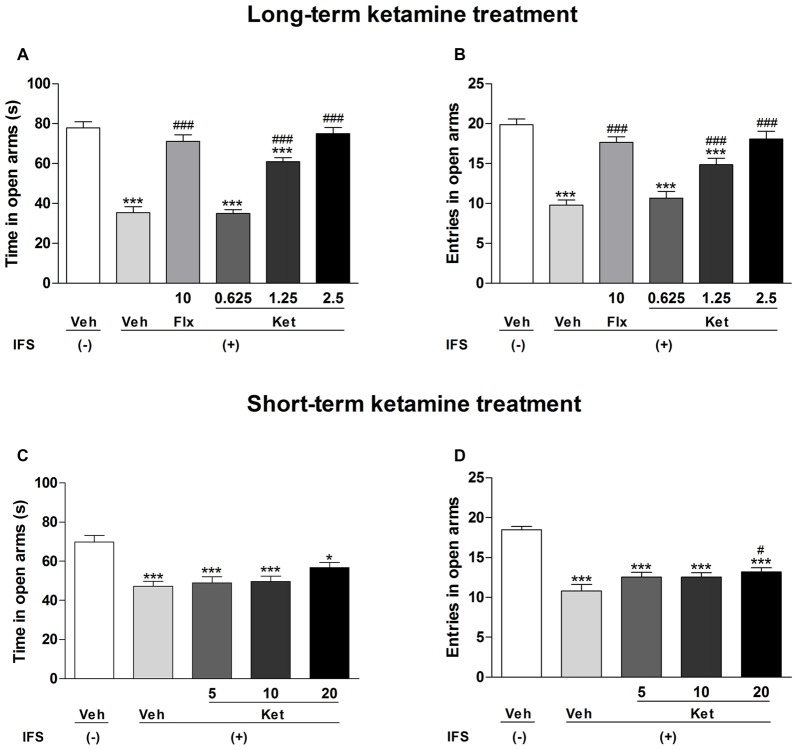
**The effects of ketamine on EPM test in IFS mice. (A,B)** Exposure to IFS resulted in a decreased percentage of time spent in and entries into open arms, whereas long-term ketamine treatment alleviated the behavioral deficits. **(C,D)** Short-term ketamine administration did not diminish the decreased percentage of time spent in and entries into open arms except the dose of 20 mg/kg ketamine. Data are presented as mean ± SEM (*n* = 15 in per group). *P < 0.05, ***P < 0.001 compared with the Veh group; ^#^*P* < 0.05, ^###^*P* < 0.001 compared with the IFS + Veh group.

We also used the EPM test to measure the anxiety-like behavior of mice. IFS animals showed significant reductions in the time spent in open arms (Long-term treatment: *F*_(5,84)_ = 49.696, *P* < 0.001, Figure 4A; short-term treatment: *F*_(4,70)_ = 10.299, *P* < 0.001, Figure 4C) and number of entries into open arms (long-term treatment: *F*_(5,84)_ = 24.416, *P* < 0.001, Figure 4B; short-term treatment: *F*_(4,70)_ = 23.306, *P* < 0.001, Figure 4D). Long-term administration of ketamine (1.25 mg/kg and 2.5 mg/kg) significantly increased the percentage of time spent in the open arms (*P* < 0.001, Figure 4A) and the number of entries into the open arms (*P* < 0.001, Figure 4B), as did the oral administrations of Flx (*P* < 0.001, Figures 4A,B). In the short-term treatment, only the dose of 20 mg/kg ketamine increased the entries into the open arms (*P* = 0.048, Figure 4D) as compared with the IFS + Veh group.

### Long-term Rather than Short-term Ketamine Treatment after Fear-conditioning Led to Fear Erasure When Combined with Extinction Training

**Figure 5:**
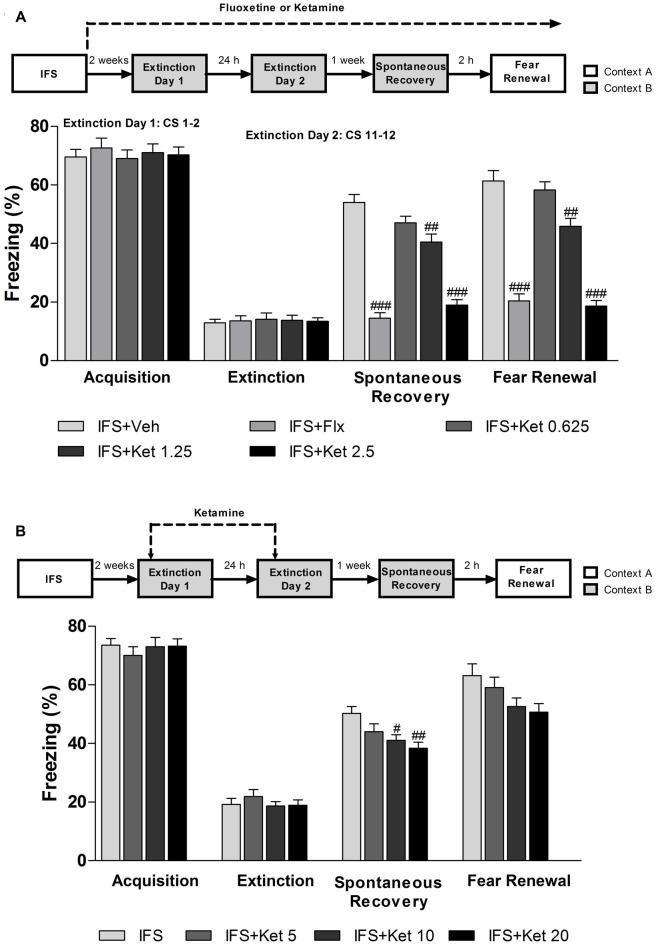
**The effect of ketamine treatment on the fear erasure when combined with extinction training. (A)** After IFS, the mice were treated with Flx or ketamine, and continued throughout the experiment. After 2 weeks of Flx or ketamine treatment, mice were subjected extinction training. A week later, spontaneous recovery of fear and renewal were performed to explore whether this fear reduction was permanent. All the groups exhibited similar levels of fear acquisition (extinction day 1, first 2 CS) and extinction (extinction day 2, last 2 CS). One week later, only IFS + Veh and IFS + 0.625 mg/kg ketamine groups showed elevated spontaneous recovery and significant fear renewal. The IFS + Flx and IFS + 1.25 and IFS + 2.5 mg/kg ketamine groups froze less than the IFS + Veh group. **(B)** Ketamine (5, 10, or 20 mg/kg) treatment started 2 h prior to the extinction training. The four groups exhibited similar levels of fear acquisition and extinction. One week later only IFS + 10 and IFS + 20 mg/kg ketamine groups froze less than the IFS + Veh group in the spontaneous recovery. All the groups showed the fear relapse. Data are presented as mean ± SEM (*n* = 15 in per group). ^#^*P* < 0.05, ^##^*P* < 0.01, ^###^*P* < 0.001 compared with the IFS + Veh group.

After successfully building the IFS model, the long-term ketamine treatment mice were given ketamine for 2 weeks, then all the groups were exposed to extinction training. Both Flx and ketamine-treated acquired extinction (*F*_(4,70)_ = 0.225, *P* = 0.924, Figure 5A). One week after the end of the extinction training, IFS mice showed significant fear response in spontaneous recovery test. However, the fear recovery was attenuated in mice treated with Flx (*P* < 0.001, Figure 5A) or ketamine (Ketamine (1.25 mg/kg): *P* < 0.01; ketamine (2.5 mg/kg): *P* < 0.001, Figure 5A) compared with the IFS + Veh group. We also performed fear renewal as an additional test to ensure that the phenomenon of fear erasure was lasting. Both the IFS + Flx group and IFS + Ket group showed attenuated fear renewal (Flx: *P* < 0.001; ketamine (1.25 mg/kg): *P* < 0.01; ketamine (2.5 mg/kg): *P* < 0.001, Figure 5A). Short-term ketamine did not influence fear acquisition. Whereas the IFS mice showed a tendency to spontaneous recovery and clear fear renewal, short-term ketamine treatment did not ameliorate the fear return in mice after extinction training (Figure 5B).

Based on these results, we only measured the biochemical markers for the long-term ketamine treatment groups.

### Long-term Ketamine Treatment Prevented the Up-regulation of the DNA Methyltransferases (DNMTs) Levels in the mPFC and HIP Induced by Foot Shocks

**Figure 6:**
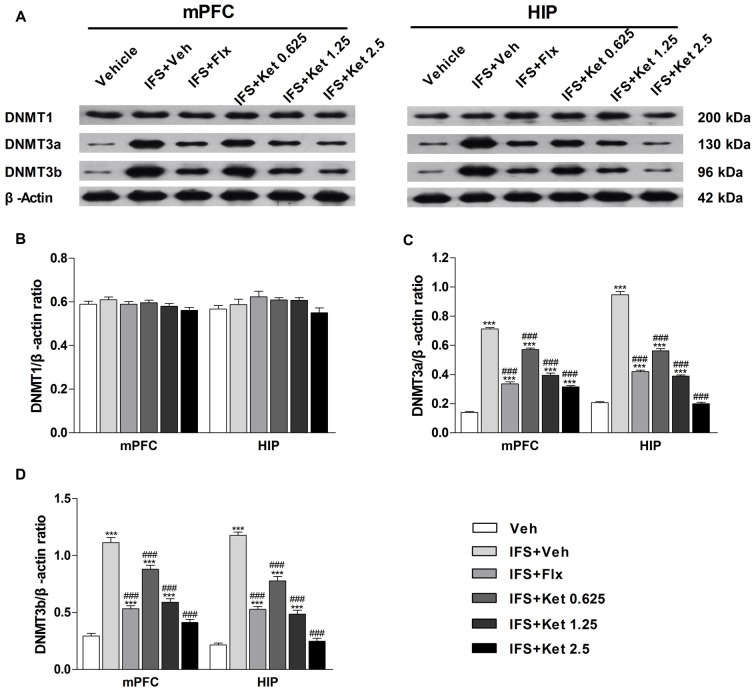
**Effects of ketamine on the levels of DNA methyltransferases (DNMTs) in the medial prefrontal cortex (mPFC) and hippocampus (HIP) of IFS mice. (A)** Representative blots of the protein levels in the six groups.** (B)** The DNMT1 had no significant difference among the six groups. **(C,D)** IFS led to the overexpression of DNMT3a and DNMT3b in the mPFC and HIP. Data are presented as mean ± SEM (*n* = 5 in per group). ***P < 0.001 compared with the Veh group; ^###^*P* < 0.001 compared with the IFS + Veh group.

To determine whether DNA methylation underlies the effects of a combination of ketamine and extinction, we examined the DNMTs in the mPFC and HIP. Our results did not show significant differences in the DNMT1 expression among the six groups (mPFC: *F*_(5, 24)_ = 1.604, *P* = 0.197; HIP: *F*_(5,24)_ = 1.919, *P* = 0.128, Figures 6A,B). Foot shocks procedure significantly increased the levels of DNMT3a and DNMT3b (*P* < 0.001, Figures 6A,C,D). This effect was reversed by the long-term administration of ketamine. A similar effect was also observed with the Flx treatment.

### Long-term Ketamine Combined with Extinction Training Promoted Fear Erasure by Regulation of Bdnf Gene Expression in the mPFC and HIP

**Figure 7:**
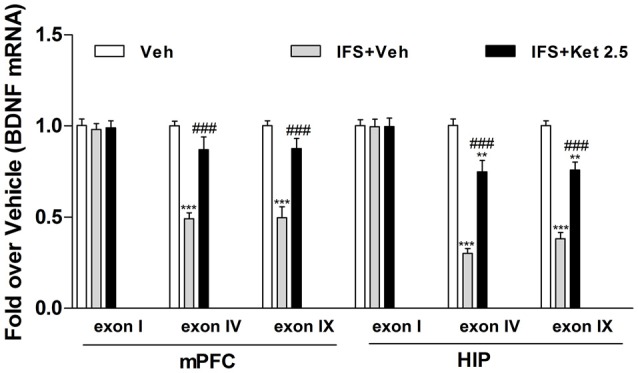
**Effects of ketamine on the level of brain-derived neurotrophic factor (BDNF) mRNA in the mPFC and HIP of IFS mice**. IFS mice showed a decrease in Bdnf transcript covering its neuronal-specific promoter (exon IV) as well as in Bdnf transcript covering its protein-coding region (exon IX) in the mPFC and HIP. Long-term ketamine treatment increased the expression of exon-specific BDNF mRNA. Data are presented as mean ± SEM (*n* = 5 in per group). **P < 0.01, ***P < 0.001 compared with the Veh group; ^###^*P* < 0.001 compared with the IFS + Veh group.

The Bdnf gene structure is complex, with multiple initiation start sites allowing exon-specific transcription of multiple mRNA transcripts. We therefore investigated whether alterations of exon-specific transcription at the Bdnf gene locus is involved in the fear erasure process. There were no significant changes in exon I mRNA level in the mPFC and HIP after the fear-conditioning and extinction program. However, exon IV and IX mRNA levels were decreased relative to the Veh group (mPFC: *F*_(2,12)_ = 32.43, *P* < 0.001; HIP: *F*_(2,12)_ = 65.84, *P* < 0.001, Figure 7). Ketamine treatment after fear-conditioning significantly increased Bdnf IV and IX mRNA levels in the mPFC and HIP (*P* < 0.001, Figure 7) compared with the IFS + Veh group.

### DNA Methylation Changes at the Bdnf Exon IV after the Combination Treatment of Ketamine and Extinction Training in the PTSD Model Mice

**Figure 8:**
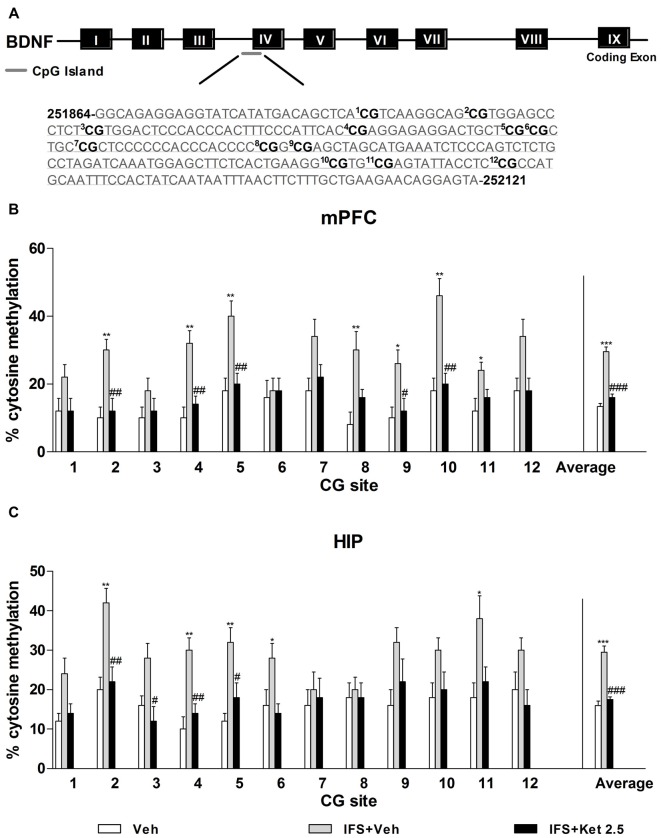
**Ketamine on the Bdnf DNA methylation in the IFS mice. (A)** Bisulfite sequencing analysis performed on 12 CpG sites near the transcription initiation site of Bdnf exon IV show percentage of cytosine residues that were methylated with fear- conditioning.** (B,C)** IFS mice displayed higher percentage of methyl cytosine residues at specific CpG sites in the mPFC and HIP, which were demethylated after ketamine treatment. Data are presented as mean ± SEM (*n* = 5 in per group). *P < 0.05, **P < 0.01, ***P < 0.001 compared with the Veh group; ^#^*P* < 0.05, ^##^*P* < 0.01, ^###^*P* < 0.001 compared with the IFS + Veh group.

Our observations of the increased levels of DNMTs and fear conditioning-associated regulation of exon-specific Bdnf transcripts led us to speculate that CpG island sites methylation of the Bdnf gene might be a mechanism mediating Bdnf gene expression in the mPFC and HIP during fear erasure. We used direct bisulfite sequencing PCR (BSP) to examine site-specific methylation within the exon IV region. A schematic of the 12 CpG dinucleotides within the exon IV region is shown in Figure 8A. We observed prominent increases in cytosine residues methylated at CpG sites 2, 4, 5, 8, 9, 10 and 12 in the mPFC (*P* < 0.05, Figure 8B) and at CpG sites 2, 4, 5, 6 and 11 in the HIP (*P* < 0.05, Figure 8C) after fear relapse. The percentage of methylated cytosine residues of Bdnf exon IV significantly decreased with ketamine treatment (*P* < 0.05, Figures 8B,C).

## Discussion

Our study provides experimental evidence that the long-term (22 days) treatment with ketamine in combination with extinction training may produce the suppression of IFS-induced anxiety-like behavior and fear relapse in mice, and the effects were similar to those induced by Flx. Furthermore, the ketamine-involved treatment increased the levels of BDNF in the mPFC and HIP of IFS mice through down-regulation of the methylation of Bdnf exon IV. These findings suggest that mechanisms underlying treatment of fear symptoms in the murine model of PTSD by ketamine in combination with extinction training involve normalization of methylation of Bdnf gene.

Studies investigating the anxiety-related effects of ketamine in animal models of PTSD are controversial, with reports of anxiolytic (Amann et al., 2009; Brachman et al., 2015; Zhang et al., 2015), no effect (Hayase et al., 2006; Groeber Travis et al., 2015), or anxiogenic (Babar et al., 2001; Juven-Wetzler et al., 2014). Such discrepancy may be attributed to a wide range of experimental designs with variable translational relevance to PTSD, including different behavioral procedures and doses routes, and durations of ketamine administration.

We found that fear relapsed during the spontaneous recovery and in response to fear renewal. Because freezing was the same in the conditioning context as it was 2 h before in the extinction context, it is impossible to confidently conclude that the cause of the return of fear was renewal. Hence, these findings should be interpreted as the evidence of fear return obtained using two different tests. The similar approach was used by other investigators (Karpova et al., 2011; Yang et al., 2016).

Pharmacological interventions to facilitate extinction memory represent promising approach to achieve lasting suppression of conditional fear. In practice, it has been demonstrated that specific histone deacetylase (HDAC) inhibitors can facilitate the consolidation of fear extinction (Stafford et al., 2012; Whittle et al., 2013; Bowers et al., 2015). Furthermore, HDAC2-targeted inhibitors coupled with memory reactivation and extinction could lead to persistent loss of even remote fear memories (Gräff et al., 2014). Similarly, numerous studies in animals and humans demonstrated that changes in DNA methylation may be involved in mediation of early symptoms of PTSD and these changes in DNA methylation may confer vulnerability to subsequent life adversity (Murgatroyd et al., 2009; Provençal et al., 2012). Consistent with these findings, our study revealed that the long-term treatment with ketamine in combination with extinction training may inhibit fear memories returning by normalizing methylation of Bdnf gene.

BDNF is one of the most studied signal transmitters in the fear network (Andero and Ressler, 2012). The Bdnf gene in rodents has at least nine 5′ non-coding exons each containing a unique promoter region and a 3′ coding exon (IX), which codes for the BDNF prepropeptide (Musumeci and Minichiello, 2011). BDNF infusion into PFC accelerated fear extinction in rats (Peters et al., 2010), whereas both humans and mice carrying the met allele of the BDNF Val66Met polymorphism show impaired extinction (Soliman et al., 2010). Clinical and preclinical studies have reported that ketamine could increase BDNF, and that BDNF and its receptor tropomyosin-related kinase B (TrkB) may have a role in the antidepressant-like activity of ketamine (Autry et al., 2011; Haile et al., 2014; Lepack et al., 2014). Our recently published study also demonstrated that administration of ketamine causes an increase in BDNF protein levels (Liu et al., 2015; Sun et al., 2016). Coincident with previous findings, our current study revealed that long-term ketamine treatment increased BDNF expression in the mPFC and HIP. It has been recently reported that epigenetic regulation of Bdnf gene could be crucial in depression (Fabbri et al., 2016). Specifically, DNA methylation of the CpG island at the promoter of the Bdnf gene might be a biological marker of depression (Fuchikami et al., 2011). Animal models of PTSD suggest that epigenetic regulation of the Bdnf gene may also be crucial for this disorder. In our study, we first found that ketamine combined with extinction could accelerate fear erasure, eliciting an increase in Bdnf exon IV mRNA and hypo-methylation of the CpG island at the Bdnf exon IV.

The brain structures, such as PFC, and the HIP are thought to be critically involved in the pathophysiology of fear extinction. BDNF-containing inputs from the hippocampal CA1 to the infralimbic cortex may mediate the efficacy of fear extinction (Heldt et al., 2007; Peters et al., 2010). In rodents, damage to the mPFC interferes with extinction, as does pharmacological disruption of memory storage in the mPFC. However, BDNF signaling in other brain regions, such as the basolateral amygdale, may also be important for fear reduction by extinction (Sotres-Bayon et al., 2004; Chhatwal et al., 2006; Karpova et al., 2011). Hence, it will be important to investigate the role of the amygdala and other brain regions in therapeutic effects of ketamine in combination with extinction training against PTSD.

The limitations of this study are as follows: first, although both Bdnf exon IV and IX mRNA levels were down-regulated in response to IFS in our study, accumulating evidence demonstrates Bdnf exon IV being involved in the formation and maintenance of extinction memories (Akirav et al., 2006; Bredy et al., 2007), we therefore only investigated the methylation of Bdnf exon IV in this study. Surely, future studies are still needed to investigate the alterations of methylation of Bdnf exon IX during fear relapse. Second, we focused on the combination effects of ketamine and extinction training, whether ketamine alone, independent of extinction training, can prevent the fear relapse, needs to be determined in our future studies.

In conclusion, this study demonstrated that loss of fearful memories could be induced through a combination of long-term treatment with ketamine in combination with extinction training. Our results provide experimental evidence that treatment with ketamine in combination with extinction training may induce erasure of conditioned fear in the mouse model of PTSD by up-regulating BDNF through the hypo-methylation of Bdnf exon IV and, increased BDNF mRNA transcription.

## Author Contributions

Jiao-JiaoY, L-SJ, Jian-JunY and M-HJ: conception and design; L-SJ, Jian-JunY, LL, J-YX, DL and M-HJ: acquisition of data and analysis; Jiao-JiaoY, L-SJ, Jian-JunY and M-HJ: interpretation of data; Jiao-JiaoY, L-SJ, Jian-JunY, LL, J-YX, DL, M-HJ and AEM: drafting and critically revised the manuscript for important intellectual content.

## Conflict of Interest Statement

The authors declare that the research was conducted in the absence of any commercial or financial relationships that could be construed as a potential conflict of interest.
